# Endocrine disrupting chemical Bisphenol A and its association with cancer mortality: a prospective cohort study of NHANES

**DOI:** 10.3389/fpubh.2024.1341789

**Published:** 2024-02-23

**Authors:** Ying Yuan, Qian Chen, Xiaorong Ding, Qin Zhong, Xiaomin Zhong

**Affiliations:** Department of Oncology, The Affiliated Huaian No.1 People’s Hospital of Nanjing Medical University, Huai’an, China

**Keywords:** environmental phenols, Bisphenol A, cancer mortality, all-cause mortality, NHANES

## Abstract

**Introduction:**

There is evidence suggesting that Bisphenol A (BPA) is associated with increased all-cause mortality in adults. However, the specific nature of the relationship between BPA exposure and cancer mortality remains relatively unexplored.

**Methods:**

The National Health and Nutrition Examination Survey (NHANES) dataset was used to recruit participants. Urinary BPA was assessed using liquid chromatography-mass spectrum (LC–MS). Through the use of multivariable Cox proportional hazard regressions and constrained cubic splines, the relationships between urine BPA and death from all causes and cancer were investigated.

**Results:**

This study has a total of 8,035 participants, and 137 died from cancers after a 7.5-year follow-up. The median level of BPA was 2.0 g/mL. Urinary BPA levels were not independently associated with all-cause mortality. For cancer mortality, the second quartile’s multivariable-adjusted hazard ratio was 0.51 (95% confidence interval: 0.30 to 0.86; *p* = 0.011) compared to the lowest quartile. The restricted cubic splines showed that the association was nonlinear (p for nonlinearity = 0.028) and the inflection point was 1.99 ng/mL.

**Conclusion:**

Urinary BPA exposure was U-shaped associated with the risk of cancer mortality, and a lower level of BPA less than 1.99 ng/mL was associated with a higher risk of cancer mortality.

## Introduction

Bisphenol A (BPA) is a kind of environmental phenols utilized in baby bottles, food containers, and dentistry (1). The exposure of humans to BPA is pervasive, originating from various sources such as consumer products, food, water, and dust (2). National biological monitoring data in the United States reveals that BPA is detectable in more than 90% of urine samples in the general population (3). Currently, 12 states have enforced regulations to restrict the use of BPA in the United States. While BPA is known to undergo rapid metabolism and is primarily eliminated through urine, its cumulative exposure in everyday items could lead to concerns regarding potential long-term health consequences (4). The potential pathways underlying BPA-induced adverse health outcomes include endocrine disruption (5), oxidative stress (6) and inflammation (7).

Exposure to bisphenol A starts very early in life, causing adverse health outcomes not only in children but also later in life (8, 9). The BPA exposure has been linked to disruptions in endocrine function and metabolism (10), which can contribute to the development of metabolic disorders (11). Some studies showed that BPA exacerbated inflammation by regulating gut physiology (12) and was involved in the development of type 2 diabetes mellitus (13), obesity (14), hypertension (15) and cardiovascular disease (16). Despite mounting evidence indicating potential toxic effects of BPA on various human cancers (17, 18), the relationship between BPA exposure and mortality remains unclear.

A study reported that BPA exposure was positively related to all-cause mortality in adults. However, nonsignificant association between BPA exposure and cancer mortality was found (19). Even so, the previous study had a lower number of sample and lacked a nonlinear analysis. Therefore, we conducted a study utilizing a comprehensive database to examine the correlation between urinary BPA levels and mortality rates related to all causes and cancer.

## Methods

### Study participants

Our study utilized data from NHANES, a program specifically designed to evaluate the health and nutritional status of individuals, both adults and children, in the United States. The data covered the period from 2003 to 2012. Individuals with missing data on urinary creatinine (*n* = 2) or mortality (*n* = 13) as well as those who were diagnosed with cancer (*n* = 68) were excluded from a pool of adult participants with complete records of urinary BPA (*n* = 8,118). Ultimately, a total of 8,035 participants were included in the study. The study was approved by the institutional review board of National Center of Health Statistics and all participants provided written informed consent.

### Covariates collection

The method for measuring baseline urinary BPA levels in NHANES has been previously described (20). In brief, spot urine samples were collected from each participant and promptly transferred to specimen containers within 4 hours of collection. The determination of urinary BPA levels involved the use of online solid phase extraction, advanced liquid chromatography, and tandem mass spectrometry. It’s worth noting that the limit of detection (LOD) for urinary BPA levels was 0.20 ng/mL. Urinary BPA levels that fell below the LOD were recorded as the LOD value divided by the square root of two. Creatinine levels were measured using the Jaffe rate reaction assay.

Furthermore, essential participant information through questionnaires, which encompassed sociodemographic details and lifestyle factors were collected. Sociodemographic variables included age, gender, race, and educational level. Race was categorized into four groups: Mexican-American, non-Hispanic white, non-Hispanic black, and other races. Educational level was divided into three categories: college or higher education, high school or equivalent, and less than high school. Lifestyle factors encompassed smoking, alcohol consumption, and physical activity level. Smoking status was determined by whether participants had smoked at least 100 cigarettes in their lifetime. Alcohol consumption was assessed based on the daily or yearly number of drinks consumed. Physical activity level was calculated using total metabolic equivalent of task minutes per week and classified into three groups: inactive, moderate, and vigorous. Body Mass Index (BMI) was computed by dividing weight (in kilograms) by the square of height (in meters). Diabetes was defined as a previously diagnosis, fasting glucose levels of ≥7.0 mmol/L, glycated hemoglobin levels of ≥6.5%, or the use of antidiabetic medication. Participants were also queried about their history of congestive heart failure, coronary artery disease, angina, heart attack, or stroke, and those who reported such conditions were identified as having a history of cardiovascular disease (CVD).

The study outcomes consisted of all-cause mortality and cancer mortality. Mortality status of the study participants was determined by linking to the National Death Index until 31st December 2015. Cancer diagnosis was according to ICD-10 codes C00-C97, which specifically identify malignant neoplasms.

### Statistical analysis

To account for selection variations, oversampling and adjustments for non-responses, the sampling weights common to NHANES data were incorporated in our study. To evaluate the variances between groups, either Student’s t-test for continuous variables or Chi-square tests for categorical variables was used. For the analysis of survival rates, univariate analysis was conducted using Kaplan–Meier analysis with the Log-rank test. Multivariable survival analysis, on the other hand, was performed using Cox proportional hazards analysis. It presented the cumulative incidence function of cancer mortality with non-cancer mortality as a competing risk. Model 1 was adjusted for urinary creatinine, Model 2 was adjusted for urinary creatinine, age, gender, and race. Model 3 was additionally adjusted for education level, BMI, drinker, smoker, activity, diabetes, and cardiovascular diseases (CVD). Restricted cubic splines with knots placed at the 5th, 50th, and 95th percentiles were used to assess potential nonlinear relationships. All statistical analyzes were carried out using R software, specifically version 3.6.

## Results

The study included a sizable cohort of 8,035 individuals, predominantly middle-aged, with a slight majority being male. According to Table 1, 137 cases of cancer mortality were documented over a 7.5-year follow-up period. The measurement of urinary bisphenol A (BPA) levels revealed a median concentration of 2.0 ng/mL. Notably, individuals who succumbed to cancer during the follow-up period tended to be older (*p* = 0.001), male (*p* = 0.035), and had higher incidences of diabetes (*p* = 0.001) and cardiovascular disease (*p* < 0.001). These observations underline the importance of considering demographic and health-related factors when assessing mortality risk.

**Table 1:** Characteristics of the study population.

Variable	Overall (*n* = 8,035)	Survivors (*n* = 7,898)	Non-survivors (*n* = 137)	*p* value
Age, years	45.9 (18.7)	45.6 (18.6)	65.4 (14.9)	<0.001
Male, %	4,120 (51.3)	4,037 (51.1)	83 (60.6)	0.035
Race, %				0.142
Non-Hispanic white	3,509 (43.7)	3,447 (43.6)	62 (45.3)	
Non-Hispanic black	1828 (22.8)	1792 (22.7)	36 (26.3)	
Mexican American	1,432 (17.8)	1,405 (17.8)	27 (19.7)	
Others	1,266 (15.8)	1,254 (15.9)	12 (8.8)	
Education, %				0.001
Less than high school	2,156 (26.8)	2,109 (26.7)	52 (38.0)	
High school or equivalent	1904 (23.7)	1864 (23.6)	35 (25.5)	
College or above	3,975 (49.5)	3,925 (49.7)	50 (36.5)	
BMI, kg/m^2^	28.7 (6.9)	28.7 (6.9)	29.0 (7.3)	0.567
Drinker (%)	4,635 (57.7)	4,479 (56.7)	104 (75.9)	<0.001
Smoker, %				0.507
Never	5,567 (69.3)	5,496 (69.6)	89 (65.0)	
Past	459 (5.7)	405 (5.1)	8 (5.8)	
Current	2009 (25.0)	1997 (25.3)	40 (29.2)	
Activity, %				0.001
Inactive	1702 (21.2)	1,695 (21.5)	22 (16.1)	
Moderate	3,362 (41.8)	3,295 (41.7)	79 (57.7)	
Vigorous	2,971 (37.0)	2,908 (36.8)	36 (26.3)	
Diabetes, %	1,137 (14.2)	1,104 (14.0)	33 (24.1)	0.001
CVD, %	751 (9.3)	720 (9.1)	31 (22.6)	<0.001
Urinary creatinine, mg/dL	117 [68, 175]	117 [68, 175]	105 [62, 173]	0.228
Bisphenol A, ng/mL	2.0 [0.9, 4.0]	2.0 [0.9, 4.0]	1.9 [0.7, 4.3]	0.412
Benzophenone-3, ng/mL	12.2 [3.7, 55.7]	13.2 [4.0, 60.7]	5.35 [1.6, 19.3]	<0.001
Triclosan, ng/mL	10.3 [2.5, 57.0]	10.9 [2.7, 58.7]	5.7 [1.6, 30.4]	<0.001

**Figure 1:**
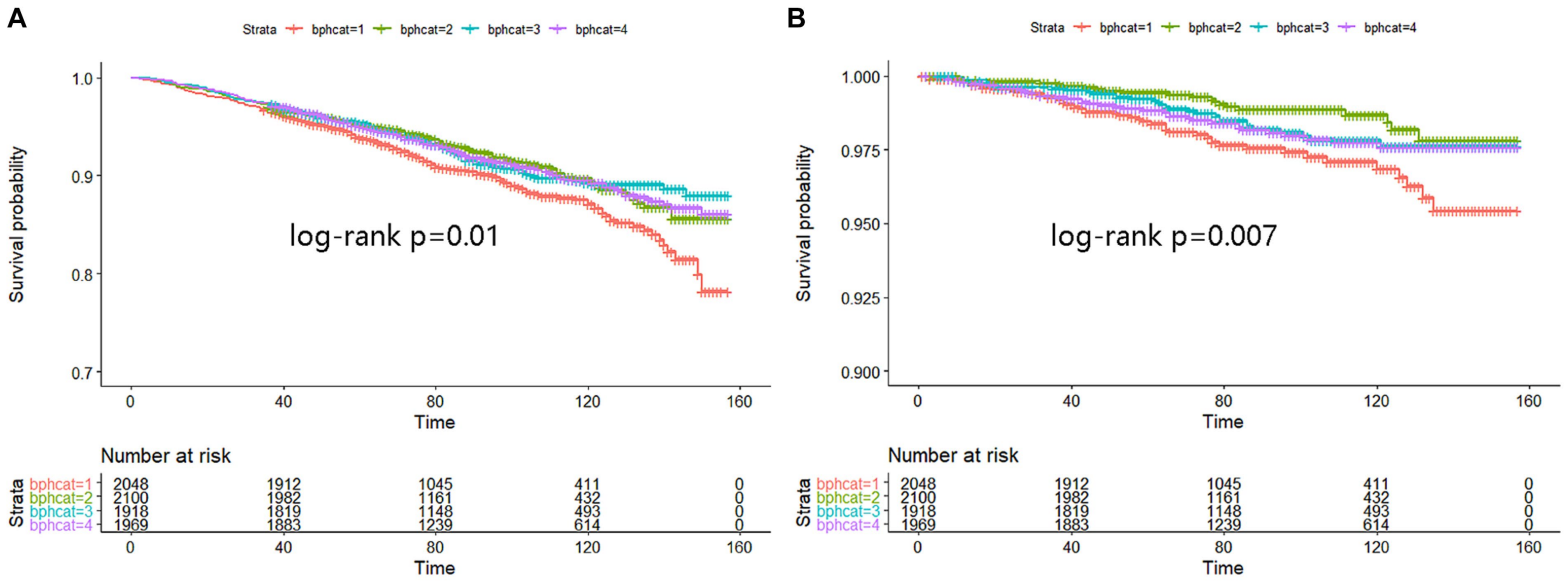
Kaplan–Meier analysis was performed to assess the relationship between urinary BPA levels and both all-cause mortality **(A)** and cancer mortality **(B)**. The X-axis represented months while Y-axis represented survival probability. Strata 1: <0.9; Strata 2: 0.9 ~ 1.9; Strata 3: 2.0 ~ 4.0; Strata 4: >4.0.

Kaplan–Meier analysis indicated significant associations between urinary BPA levels and both all-cause mortality and cancer mortality. The analysis suggests that lower urinary BPA levels are associated with an increased risk of all-cause mortality (log-rank *p* = 0.01) and cancer mortality (log-rank *p* = 0.007) (Figure 1). However, compared with the lowest quartile of BPA, no association of all-cause mortality was observed in any quartiles across models, which suggested that BPA was not independently associated with all-cause mortality (Table 2).

**Table 2:** Adjusted hazard ratios for associations between BPA and all-cause mortality.

Bisphenol A, ng/mL	Model 1		Model 2		Model 3	
	HR	*p*	HR	*p*	HR	*p*
<0.9	Ref	–	Ref	–	Ref	–
0.9 ~ 1.9	0.88 [0.71, 1.09]	0.254	0.88 [0.71, 1.09]	0.249	0.88 [0.71, 1.09]	0.246
2.0 ~ 4.0	0.98 [0.78, 1.23]	0.841	1.07 [0.86, 1.34]	0.536	1.07 [0.86, 1.34]	0.530
>4.0	1.11 [0.87, 1.41]	0.394	1.11 [0.88, 1.39]	0.381	1.02 [0.81, 1.28]	0.887

**Figure 2:**
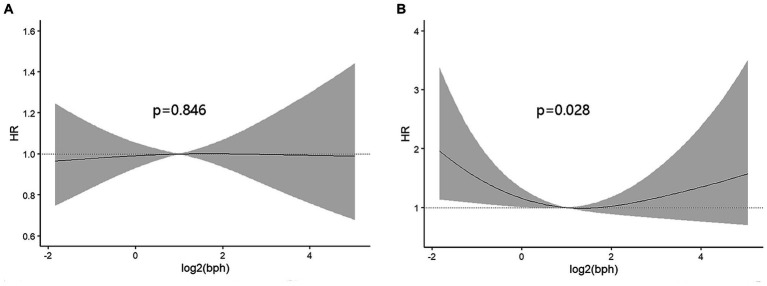
The dose–response analysis was performed to assess the relationship between urinary BPA and all-cause mortality **(A)** or cancer mortality **(B)**. The X-axis represented log-transformed BPA concentrations while Y-axis represented hazard ratio.

On the contrary with expectations, the risk of cancer mortality was reduced with the increase of BPA levels both in the unadjusted model and adjusted models (Table 3). Most importantly, the second quartile’s multivariable-adjusted hazards ratio (HR) was 0.51 (95% CI: 0.30 to 0.86; *p* = 0.011) compared to the lowest quartile of BPA. However, these associations were not consistent across all quartiles of BPA levels, indicating a more nonlinear relationship. In order to confirm the nonlinear relationship, we used restricted cubic splines (Figure 2). We found that urinary BPA was U-shaped associated with cancer mortality (p for nonlinearity = 0.028). This suggests that the relationship is not purely linear, but rather exhibits a threshold effect. Specifically, lower levels of BPA (below approximately 1.99 ng/mL) were associated with an increased risk of cancer mortality. Beyond this threshold, higher BPA levels appeared to confer protection against cancer mortality. This nonlinear relationship highlights the complexity of BPA’s impact on health outcomes and underscores the need for careful consideration of dose–response relationships. Future research should focus on elucidating the mechanisms underlying these observed associations and conducting longitudinal studies to validate these findings across diverse populations and settings.

**Table 3:** Adjusted hazard ratios for associations between BPA and cancer mortality.

Bisphenol A, ng/mL	Model 1		Model 2		Model 3	
	HR	*p*	HR	*p*	HR	*p*
<0.9	Ref	–	Ref	–	Ref	–
0.9 ~ 1.9	0.44 [0.26, 0.74]	0.002	0.46 [0.27, 0.77]	0.003	0.51 [0.30, 0.86]	0.011
2.0 ~ 4.0	0.66 [0.40, 1.08]	0.099	0.76 [0.47, 1.23]	0.264	0.77 [0.47, 1.26]	0.304
>4.0	0.72 [0.43, 1.22]	0.221	0.79 [0.48, 1.29]	0.342	0.81 [0.48, 1.36]	0.426

## Discussion

In this study, we found that urinary BPA was U-shaped associated with the risk of cancer mortality and a lower level less than 1.99 ng/mL increasing the risk of cancer mortality. This study provides valuable insights into the complex relationship between urinary BPA levels and cancer mortality, emphasizing the need for a nuanced understanding of dose–response dynamics and the consideration of confounding factors in epidemiological research.

BPA is a widely used raw material and is involved in the initiation and development of hormone-dependent cancers. A comparable study discovered that higher exposure to BPA was independently related with an elevated risk of all-cause mortality but with no significant association with cancer mortality (19). They divided BPA into tertiles and did not explore the nonlinear relationship. A recent study showed that the highest tertile of urinary BPA levels corresponded to a 36% increase in all-cause mortality and a 62% increase in CVD mortality compared to the lowest tertile (21). Different from previous study, we demonstrated a U-shaped association between BPA exposure and cancer mortality using dose–response analysis and adjusting for more variables. A study also found that BPA was not significantly associated with all-cause mortality in overall population, but in the obesity, diabetes, and hypertension subgroups (22). Variations in the characteristics of the study populations, especially comorbidity, may account for the discrepancy.

BPA is an endocrine disruptor with multiple effects. BPA exhibits estrogenic properties by binding to estrogen receptors and interfering with the regular functioning of the endocrine system (23). Additionally, BPA has the potential to influence biological processes, including cell signaling, gene expression, and apoptosis, which can contribute to a range of health issues affecting the reproductive, immune, metabolic, and nervous systems (24, 25). Several studies found a U-shaped association between BPA levels and the risk of diabetes (26) and obesity (27). A higher level of BPA impacted the production of ROS (6), cancer metabolites (28), and tumoral immune microenvironment (29), contributing to the migration and invasion of cancer cells. Conversely, a lower level may be the reflection of an imbalance of endocrine-related pathways. BPA could inhibit DNA replication and cell proliferation in tumor cells (30) by modulating cell cycle-and apoptosis-related proteins and genes in cancerous cells (31). Therefore, a lower and higher level of BPA both influenced the cancer mortality. More studies are warranted to explain the dose-repose relationship.

**Figure 3:**
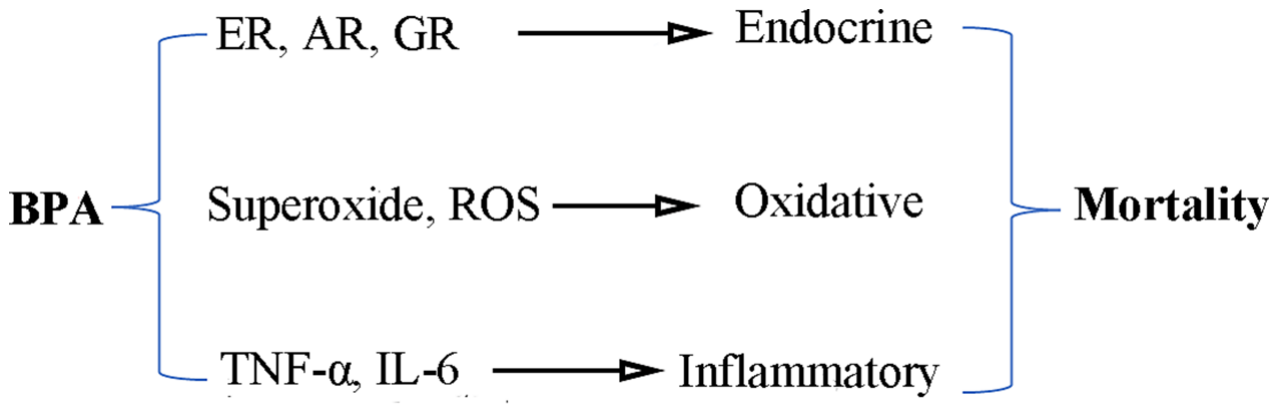
The pathophysiological mechanisms between BPA and cancer mortality. ER, estrogen; AR, androgen; GR, glucocorticoid; ROS, reactive oxidative stress.

There may be various underlying mechanisms driving the positive correlation between BPA and all-cause mortality (Figure 3). Firstly, BPA caused endocrine disruption through agonistic or antagonistic behavior at various nuclear receptors such as estrogen (ER), androgen (AR) and glucocorticoid (GR) (32). Besides, BPA exposure resulted in a strong induction of oxidative stress and inflammatory response (33, 34). However, more research is necessary to elucidate the biological mechanisms underlying this association.

This study boasts several notable strengths, including its use of a nationally representative cohort from the United States and rigorous quality control measures. Nonetheless, this study does come with certain constraints. To begin with, BPA levels were assessed solely through spot urine samples at the baseline, offering no insight into long-term BPA exposure or fluctuations in BPA concentrations within the body. Lastly, it’s important to note that the generalizability of this study could be limited due to the exclusion of participants with incomplete covariate data, potentially introducing selection bias. BPA exposure is more related to hormone-associated cancers such as breast, prostate and ovarian cancers. However, the number of specific cancer type was few in the original database, which need to be verified by further large-scale cancer epidemiological investigations.

## Conclusion

In conclusion, we found BPA exposure was U-shaped associated with the risk of cancer mortality, and a lower level of BPA less than 1.99 ng/mL was associated with a higher risk of cancer mortality. Our results can serve as valuable information for guiding policies related to enhanced monitoring of chemical exposures and risk assessment in cancer prevention field.

## Data availability statement

The raw data supporting the conclusions of this article will be made available by the authors, without undue reservation.

## Ethics statement

The studies involving humans were approved by National Center of Health Statistics. The studies were conducted in accordance with the local legislation and institutional requirements. The participants provided their written informed consent to participate in this study.

## Author contributions

YY: Writing – original draft. QC: Data curation, Writing – original draft. XD: Supervision, Writing – original draft. QZ: Project administration, Writing – review & editing. XZ: Project administration, Writing – review & editing.
